# Impact of State of Ripeness and Culinary Treatments on the Hypoglycemic, Antioxidant, and Nutritional Properties of Two Varieties of *Solanum aethiopicum* L. Fruit

**DOI:** 10.1155/ijfo/2713487

**Published:** 2026-05-05

**Authors:** Ghislain Maffo Tazoho, Josephat Gabriel Zazi Weike, Justine Odelonne Kenfack, Vanis Slauvers Akago, Donald Sévérin Dangang Bossi, Hermine Doungue Tsafack, Hilaire Macaire Womeni, Inocent Gouado

**Affiliations:** ^1^ Department of Biochemistry, University of Dschang, Dschang, Cameroon, univ-dschang.org; ^2^ Department of Animal Biology, University of Yaoundé 1, Yaoundé, Cameroon, uy1.uninet.cm; ^3^ Department of Biochemistry, University of Douala, Douala, Cameroon, univ-douala.cm

**Keywords:** antioxidant properties, culinary treatments, hypoglycemic potential, nutritional parameters, ripeness, *Solanum aethiopicum* L

## Abstract

The aim of this study was to characterize two varieties of *Solanum aethiopicum* according to their ripeness stage and culinary treatments. Green and red fruits from the local variety (V1) and the modern variety (V2) were subjected to steaming and boiling, resulting in 24 samples. These samples were used to assess the phytochemical, nutritional, antioxidant, and hypoglycemic properties of *S. aethiopicum* using standard methods. The results showed that all the samples had high fiber content, with the highest fiber content found in the pulps of each variety (26.76 ± 0.16%DM for V1 and 30.82 ± 0.10%DM for V2). The results also showed that culinary treatments reduced the antinutrient (phytates, oxalates, and tannins) content. The presence of minerals in the samples, such as iron and zinc, varied, with iron contents ranging between 2.27 ± 0.01 and 7.48 ± 0.03 mg/100 g, and zinc contents ranging between 1.02 ± 0.01 and 21.97 ± 0.32 mg/100 g. The red samples showed higher total phenol content before culinary treatment in both varieties, with a value of 101.01 ± 1.04 mg GAE/g; however, a significant decrease was observed after the two cooking methods. The samples also showed the ability to reduce Fe^3+^ to Fe^2+^ and to scavenge DPPH free radicals. The ability of *S. aethiopicum* extracts to inhibit the α‐amylase enzyme showed inhibition percentages above 50% in the selected samples in vitro. Furthermore, in vivo, the oral glucose tolerance test showed that the extracts exhibited hypoglycemic activity, as the samples effectively regulated glycemic peaks compared with the negative control. In conclusion, the steamed, skin‐on fruits of the local variety of *S. aethiopicum* exhibited the best nutritional, antioxidant, and hypoglycemic properties.

## 1. Introduction

According to the Food and Agriculture Organization of the United Nations [1], food systems refer to the processes by which humans obtain their food, transform it, consume it, and derive energy and nutrients from it to maintain their health and well‐being. However, changes in eating habits, including the consumption of fatty and sugary foods combined with a low intake of fruits and vegetables, are playing a major role in the global increase of chronic diseases, such as obesity and Type 2 diabetes [2]. Type 2 diabetes is considered a global epidemic, affecting millions of people worldwide [3]. It is responsible for approximately 9% of total mortality and causes chronic complications, such as atherosclerosis, nephropathy, and neuropathy [4]. Modern diabetes treatment uses several families of oral hypoglycemic agents, such as biguanides, thiazolidinediones, and sulfonamides [5]. However, these drugs are often expensive, not easily accessible, and can cause adverse effects [6]. Therefore, several studies have been carried out on plant‐based dietary approaches for the management of this condition. Many authors, such as Eletta et al. [7], have shown that African eggplant species (*Solanum aethiopicum*) have a high nutritional and therapeutic value and can be considered functional foods. Tuem et al. [8] revealed that *S. aethiopicum* maintained glycemia and proteinuria within normal limits. Ponticelli et al. [9] also showed that eggplants were able to inhibit the activity of certain enzymes involved in carbohydrate metabolism. Some authors [10, 11] have also shown that bioactive compounds in foods can be destroyed by heat and that cooking methods can influence their physicochemical, phytochemical, and nutritional characteristics. Solanum species belong to the family of Solanaceae with over 1000 species. Among these species, *S. aethiopicum* constitutes part of traditional sub‐Saharan African culture. Wide variations exist within and between the African eggplants species including variations in characters, such as diameter of corolla, petiole length, leaf blade width, plant branching, fruit shape, and color [12, 13]. In Cameroon, particularly in the Western region, these varieties are commonly consumed, namely the modern and the local varieties. The ethnonutritional study on *S. aethiopicum* L. fruits was done in West Cameroon, and the results obtained showed that these fruits are consumed in various ways depending on the growth stage. *S. aethiopicum* can be eaten raw and also used in the preparation of the “Yellow sauce,” “Eggplant sauce,” and “Black sauce.” It is known that when used in the preparation of different sauces, *S. aethiopicum* can be boiled or steamed to remove the very fibrous cortex [14]. In addition to the studies and issues mentioned above, the specific effects of boiling and steaming, as well as the ripeness stage of the different varieties of *S*. *aethiopicum*, on their phytochemical, antioxidant, and hypoglycemic properties have not yet been fully elucidated. The aim of this study was therefore to determine the nutritional, phytochemical, and hypoglycemic properties of two varieties of *S. aethiopicum* in relation to their ripeness stage and culinary treatments, with the objective of identifying the most suitable variety for the management of glycemic regulation in the body.

## 2. Materials and Methods

### 2.1. Plant Material

Two varieties of *S. aethiopicum* L. were harvested in the Bangang community (West Cameroon) in February 2024. The samples were collected at maturity stages 1 (green to yellowish‐green coloration) and 4 (red coloration), as described by Campos et al. [15]. They were then transported in bags to the Research Unit of Biochemistry of Medicinal Plants, Food Sciences, and Nutrition (URBPMAN) of the Department of Biochemistry at the University of Dschang for various treatments and analyses. The plant material was identified at the National Herbarium of Cameroon as *S. aethiopicum* L. (cv. group Gilo) under identification number 43012/HNC. The following varieties were used: the local variety (Figure 1(a)) and the modern variety (Figure 1(b)).

FIGURE 1(a) Local variety (V1) of *Solanum aethiopicum* L. at the green (A) and red (B) maturity stages. (b) Modern variety (V2) of *Solanum aethiopicum* L. at the green (A) and red (B) maturity stages.

**Figure:**
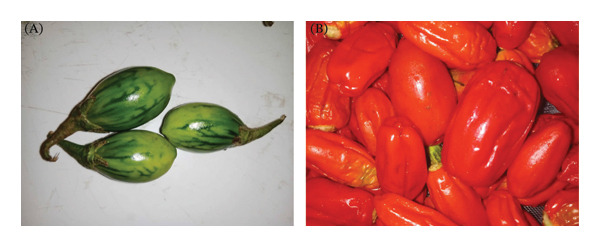
(a)

**Figure:**
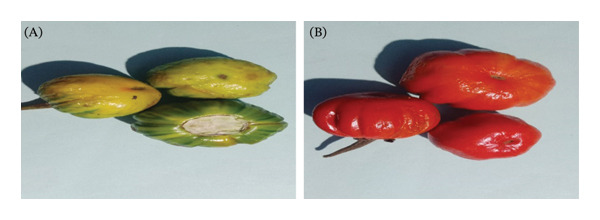
(b)

### 2.2. Culinary Processing of Samples and Production of Powders

Some of the samples were used raw, while others underwent culinary processing (boiling and steaming). The raw samples were cleaned and dried directly in an oven.

#### 2.2.1. Boiling

Two kilograms (2 kg) of each sample was cooked in water at 90°C for 30 min in a hermetically sealed stainless‐steel pot on a branded hot plate (Model: JX‐1010B). The samples were then divided into two batches. The first batch consisted of peeled (skin‐off) eggplants, and the second batch consisted of unpeeled (skin‐on) eggplants from both varieties at different ripeness stages. For the first batch, the cortex (peel) was separated from the pulp (flesh), and analyses were performed separately on each fraction (cortex and pulp/flesh). However, the cortex from boiled and steamed fruits of each variety was pooled for analysis, irrespective of maturity stage. Each treatment was carried out separately for each variety and at each maturity stage.

#### 2.2.2. Steaming

The fruit was placed on a sieve in a pot containing water and hermetically sealed so that no steam could escape for 30 min. The samples were then divided into different batches as in the boiling treatment.

#### 2.2.3. Powder Production

All the samples (raw/skin‐on; pulp and whole fruit after boiling; pulp and whole fruit after steaming; and cortex of boiled and steamed fruits) were dried in a ventilated oven at 45°C until a constant dry mass was obtained. They were then ground using an electric grinder, and the resulting powders were stored in a desiccator for subsequent analyses. A total of 24 samples were obtained, that is, 12 samples per variety. The samples were distributed as follows:

Samples before Culinary Treatments (Skin‐on Fruits): REV1 = skin‐on red fruit variety 1, VEV1 = skin‐on green fruit variety V1, REV2 = skin‐on red fruit variety 2, VEV2 = skin‐on green fruit variety 2;

pulp and whole fruit after boiling: REBV1 = skin‐on red boiled fruit variety 1, VEBV1 = skin‐on green boiled fruit variety 1, RSPBV1 = pulp of red boiled fruit variety 1, VSPBV1 = pulp of green boiled fruit variety 1, REBV2 = skin‐on red boiled fruit variety 2, VEBV2 = skin‐on green boiled fruit variety 2, RSPBV2 = pulp of boiled red fruit variety 2, VSPBV2 = pulp of boiled green fruit variety V2;

pulp and whole fruit after steaming: VEVV1 = skin‐on green steamed fruit variety 1, REVV1 = skin‐on red steamed fruit variety 1, VSPVV1 = pulp of green steamed fruit variety 1, RSPVV1 = pulp of red steamed fruit variety 1, VEVV2 = skin‐on green steamed fruit variety 2, REVV2 = skin‐on red steamed fruit variety 2, VSPVV2 = pulp of green steamed fruit variety V2, RSPVV2 = pulp of red steamed fruit variety 2.

Cortex of Boiled and Steamed Fruits (Skin‐off): PRV1 = cortex of red fruit boiled and steamed variety 1, PVV1 = cortex of green fruit boiled and steamed variety 1, PRV2 = cortex of red fruit boiled and steamed variety 2, PVV2 = cortex of green fruit boiled and steamed variety 2.

### 2.3. Aqueous Extraction of Different Samples of *S. aethiopicum* L. Powders

Five grams (5 g) of powder from each sample was macerated in 50 mL of distilled water for 48 h at room temperature. The mixture was filtered using Whatman No. 1 filter paper. After filtration, the resulting filtrate was placed in a ventilated oven at 45°C for 24 h. The percentage yield was obtained using the following formula: Yield (%) = (*W*1)/(*W*2) × 100 where *W*1 is the weight of the extract residue after solvent removal and *W*2 is the weight of dried plant powder. The obtained samples were stored in a refrigerator at 4°C for subsequent analysis [16].

### 2.4. Determination of the Antinutritional Properties of *S. aethiopicum* Powders

#### 2.4.1. Phytate Content

The method described by AOAC [17] was used to quantify the phytate content in this study. The mixture, consisting of 2 g (2 g) of sample and 100 mL of 2% HCl, was left to stand for 3 h in a 250‐mL Erlenmeyer flask. Afterward, the solution was filtered using Whatman No. 4 filter paper. Next, 107 mL of distilled water, 50 mL of the filtrate, and 10 mL of 0.3% ammonium thiocyanate (NH4SCN) as an indicator were mixed in a 250‐mL Erlenmeyer flask. A standard FeCl_3_·6H_2_O solution containing 0.00195 g of iron per milliliter was used to titrate the mixture. The color change to yellow–orange, which persisted for 5 min, served as the endpoint indicator. The amount of phytates was determined by the following formula:

(1) Phytates mg/100 g=X×1.190.00195.

With *X* corresponding to descent of the burette.

#### 2.4.2. Oxalate Content

The method described by Day et al. [18] was used to determine the oxalate content. The method employed was colorimetric titration with potassium permanganate. A volume of 75 mL of 1.5 M H_2_SO_4_ (sulfuric acid) and 1 g (1 g) of sample were placed in an Erlenmeyer flask. The mixture was stirred using a magnetic stirrer and then filtered through Whatman No. 1 filter paper. A 0.1 M potassium permanganate (KMnO_4_) solution was used at high temperature (80°C–90°C) to titrate 25 mL of the filtrate placed in a beaker until the solution developed a persistent pink color for 30 s. The oxalate content was determined using the following relationship:

(2) 0.10.00450 mL KMnO4= g oxalate.

#### 2.4.3. Saponin Content

The saponin content was determined by the method of Kozioł [19]. A volume of 5 mL of distilled water and a mass of 0.5 g of the sample were measured and placed in a test tube and shaken vigorously for 30 s. The saponin content was quantified based on the height of the foam formed immediately after 5–10 s of shaking, using the following formula: Saponins (mg/100 g) = ([0.432 × height of foam in cm] + 0.008/Mass of sample [g]).

#### 2.4.4. Condensed and Hydrolyzable Tannin Content

The method described by Luzardo‐Ocampo et al. [20] was used. Firstly, a volume of 25 mL of 1% HCl (in methanol) and 1 g (1 g) of powder were measured and introduced into a 50‐mL beaker. The mixture was stirred for 30 min and then centrifuged at 4000 rpm for 15 min, and the supernatant was collected in another beaker. The residue was collected and extracted twice. To quantify the condensed tannins, the mixture, consisting of 5 mL of the reagent solution (8 mL of hydrochloric acid in 100 mL of distilled water and 50 g of vanillin) and 1 mL of the extract, was incubated at 30°C for 20 min, and the absorbance was read at 500 nm. A standard tannic acid solution was used to calculate the tannin content in each sample. Concerning hydrolyzable tannins, the optical density of the mixture, consisting of 1.75 mL of reagent solution (0.01 M FeCl_3_·6H_2_O in 0.001 M HCl) and 500 μL of the extract, was read at 660 nm. A standard tannic acid solution was used to calculate the condensed and hydrolyzable tannin contents, and the results were expressed as mg tannic acid/100 g extract.

### 2.5. Determination of Phenolic Compounds

#### 2.5.1. Total Phenol Content

Total phenols were determined spectrophotometrically using the colorimetric method with the Folin–Ciocalteu reagent, as described by Gao et al. [21]. The absorbance of the test tube, consisting of a mixture of 1.39 mL of distilled water, 0.2 mL of Folin–Ciocalteu reagent, 0.01 mL of a 5 mg/mL extract solution, and 0.4 mL of sodium carbonate (Na_2_CO_3_, 20%), was read at 760 nm after incubation at 40°C for 20 min using a water bath. Concomitantly, a freshly prepared aqueous solution of gallic acid (0.2 g/L) was used for calibration, and the results were expressed as mg gallic acid equivalent per g extract (mg GAE/g extract).

#### 2.5.2. Total Flavonoid Content

The colorimetric method described by Bahorun et al. [22] was used to determine the flavonoid content of the extracts. Firstly, an initial mixture consisting of 0.03 mL of a 5% sodium nitrite (NaNO_2_) solution, 1.4 mL of distilled water, and 0.1 mL of extract was done. After 5 min, the addition of 0.2 mL of a 10% aluminum trichloride (AlCl_3_) solution to the first mixture was done, and the solution was left to stand for a further 5 min. Finally, 0.24 mL of distilled water and 0.2 mL of 10% concentrated NaOH solution have been added to the mixture, and the results were expressed as milligrams of catechin equivalent per gram of extract (mg CE/g extract) after measuring the absorbance of the mixture at 510 nm.

### 2.6. Determination of the Proximate Chemical Composition, Mineral Content, and Reducing Sugars of the Samples

Regarding these analyses, it is important to note that fiber, moisture, ash, dry matter, and organic matter contents were assessed on all 24 initial samples, while the others (protein, lipids, carbohydrates, minerals, reducing sugar, and color test) were performed on only 11 samples selected after principal component analysis (PCA). The fiber, protein, lipid, ash, dry matter, organic matter, and moisture contents of the samples were determined using the method of the Association of Official Analytical Chemists [17].

#### 2.6.1. Proximate Chemical Composition

Fiber Content: The mixture, consisting of 0.255 N sulfuric acid and 1 g (1 g) of each sample, was placed in a beaker and boiled for 30 min, followed by filtration using Whatman paper. A volume of 5 mL of hot water (three times) and 5 mL of acetone (twice) was used consecutively to wash the mixture after adding 0.313 N sodium hydroxide to the residue and boiling for another 30 min. Finally, the weight of the insoluble residue was measured after drying the mixture at 105°C for 8 h. The ash content was obtained after incineration of the dry residue at 550°C for 3 h, and the fiber content was calculated according to the following formula: Fibers = (M1 − M2/*M* × DM) × 100 where M1 is the mass of the sample dried at 105°C, M2 is the mass of the sample dried at 550°C, *M* is the mass of the test sample, and DM is a % dry matter.

Lipid content was determined by the Soxhlet method and protein content by the Kjeldahl method. In a Soxhlet extractor, the sample lipids were extracted in an organic solvent, followed by drying, and finally, the oil mass was calculated. Concerning the protein content, the nitrogen content of the powders was first determined by the Kjeldahl method, which involved successive mineralization, distillation, and titration. The crude protein content was obtained by multiplying the total nitrogen content of the sample by the conversion factor 6.25. The total carbohydrate content was obtained by difference using the following formula: % Carbohydrate = 100 ‐ (% Protein + % Fat + % Ash + Fiber + Moisture).

#### 2.6.2. Reducing Sugar Content

The reducing sugars contained in the samples were measured using the AACC method [23]. A mixture consisting of 10 mL of water, and 0.5 g of each sample was measured and incubated at 95°C for 15 min using a water bath. A volume of 4 mL of water was used to wash the pellet, and the Whatman No. 4 paper was used for the filtration. Finally, 0.1 mL of previously prepared zinc acetate and 0.1 mL of 10.6% ferrocyanide were added and centrifuged, and the supernatant was collected and the volume recorded. The assay was carried out by taking 125 μL of sample, 500 μL of water, and 125 μL of pre‐prepared DNS. The absorbance was read at 540 nm after heating the mixture for 15 min using water bath.

#### 2.6.3. Mineral Content

The mineral content was determined using the Pauwels et al. [24] method for sodium (Na), calcium (Ca), magnesium (Mg), phosphorus (P), zinc (Zn), potassium (K), and iron (Fe). Calcium and magnesium were determined by complexometry, using the Ca + Mg mixture on the one hand and Ca on the other. The air/propane flame photometer at 768 and 589 nm was used, respectively, to determine the Na and K contents of the samples. The concentrations of 0, 5, 10, 15, and 20 ppm and the concentrations of 0, 50, 100, 150, and 200 ppm, respectively, for sodium and potassium were used as standard solutions. The absorption spectrophotometry at 420 and 665 nm was used to determine Fe and Zn content of the samples, respectively. The standard solutions were prepared at 1, 2, 4, and 6 ppm and 0, 2.5, 5, and 10 ppm, respectively, for zinc and iron. Phosphorus is present in the extract in the form of orthophosphate. Together with the vanadate and molybdate ions, the phosphate forms a yellow phospho‐vanado‐molybdate complex that can be measured by molecular absorption spectrophotometry at 430 nm. The linear regression equation obtained from the absorbances of the different standard solutions was used to calculate the concentration of each mineral.

### 2.7. Determination of In Vitro Antioxidant Properties

#### 2.7.1. DPPH (2,2‐Diphenyl‐1‐Picrylhydrazyl) Test

The DPPH test was performed as described by Mensor et al. [25]. In the analysis protocol, 0.9 mL of DPPH (prepared in pure methanol to give a solution with a final concentration of 17 mg/mL) was introduced into a test tube containing 0.1 mL of each extract diluted to final concentrations of 200, 100, 50, 25, and 12.5 μg/mL. The mixture was then shaken well for 5 min and incubated in the dark for 30 min at room temperature (25°C). For the control tube, methanol was used instead of extract for each concentration. Readings were taken by measuring absorbance at 517 nm with a “BK‐D590” spectrophotometer. The antioxidant activity of the extracts was expressed as a percentage of inhibition according to the following equation:

(3) Anti−free radical activity %=Abs control−Abs testAbs control×100.

The equation %AA = *a* log(*C*) + *b* was used to determine the IC_50_ value (inhibitory concentration at 50) from the inhibition percentage of antioxidant action.

#### 2.7.2. Ferric Reducing Antioxidant Power (FRAP) Assay

The antioxidant activity of the different extracts was also assessed using the method for reducing ferric ion Fe^3+^ to ferrous ion Fe^2+^ [26]. To do this, a mixture consisting of 2.5 mL of a 1% potassium ferricyanide K_3_Fe(CN)_6_ solution (1 g of K_3_Fe(CN)_6_ in 100 mL of distilled water), 2.5 mL of a 0.2 M phosphate buffer solution (pH = 7.4), and 1 mL of sample at different concentrations (200, 100, 50, and 25 μg/mL) was incubated at 50°C for 20 min using water bath. Afterward, the reaction was stopped using 2.5 mL of 10% trichloroacetic acid, and the mixture was centrifuged for 10 min at 3000 rpm. Then, the absorbance of the solution consisting of 0.5 mL of a freshly prepared 0.1% ferric chloride solution, 2.5 mL of distilled water, and an aliquot of 2.5 mL of the supernatant was read at 700 nm. The control was represented by a standard solution of vitamin C, the absorbance of which was measured under the same conditions. A calibration curve was drawn of the straight line obtained with the Trolox used as a reference at different concentrations. The total reducing power was expressed in Trolox equivalent (μmol Trolox/g extract).

### 2.8. Evaluation of the Coloration of Different *S. aethiopicum* Powders

Color values *L* ^∗^, *a* ^∗^, and *b* ^∗^ (lightness, red/green value, and blue/yellow value, respectively) were measured using a HunterLab colorimeter (ColorFlex EZ model: 45/0 LAV). The colorimeter was calibrated with a white ceramic plate using the D_65_ illumination source at an angle of 10° [27]. The instrument was calibrated against a light yellow reference tile. A glass cell containing the powder was placed above the light source and covered with a white plate, and the *L* ^∗^, *a* ^∗^, and *b* ^∗^ values were recorded. The whiteness index (IB) of the flours was determined according to the following equation: IB = 100 − $\sqrt{\left(1001-\right)222+a+b}$.

### 2.9. Identification of Bioactive Compounds Using the Gas Chromatography–Mass Spectrophotometry (GC‐MS) Method

The bioactive compounds were determined by the method of El‐Naggar et al. [28], using only one sample (whole raw fruit) from each variety. A mass of 1 g of powder was extracted with methanol (5 mL) for 24 h using a cold maceration procedure on a homogenization plate. The extracts were then obtained by filtering the supernatant through Whatman filter paper containing sodium sulfate. They were subsequently processed using standard protocols for analysis by gas chromatography–mass spectrometry (GC‐MS; GC model: 8890GC/5977B GC/MSD‐Agilent), enabling the various bioactive compounds to be identified. The identity of the biological constituents was determined by comparing their retention times (RT) and the fragmentation patterns of the mass spectra with those stored in the NIST library database (Version 2020). MassHunter Workstation software was used for the analysis.

### 2.10. Determination of the Hypoglycemic Activity of *S. aethiopicum* Extracts

**FIGURE 2:**
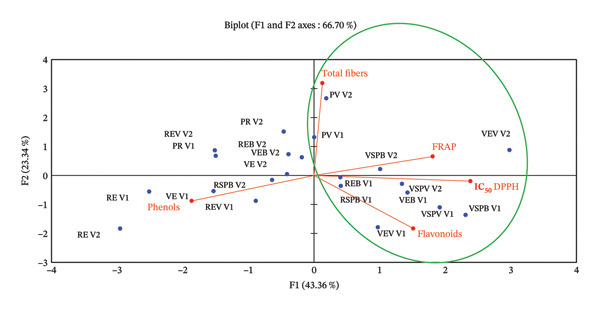
Principal component analysis of total phenols, flavonoids, IC_50_ DPPH, FRAP, and crude fiber as active variables and active observations, REV1 = skin‐on red Fruit Variety 1, VEV1 = skin‐on green Fruit Variety V1, PRV1 = cortex of red fruit boiled and steamed Variety 1, PVV1 = cortex of green fruit boiled and steamed Variety 1, REBV1 = skin‐on red boiled Fruit Variety 1, VEBV1 = skin‐on green boiled Fruit Variety 1, VEVV1 = skin‐on green steamed Fruit Variety 1, REVV1 = skin‐on red steamed Fruit Variety 1, RSPBV1 = pulp of red boiled Fruit Variety 1, VSPBV1 = pulp of green boiled Fruit Variety 1, VSPVV1 = pulp of green steamed Fruit Variety 1, RSPVV1 = pulp of red steamed fruit variety, REV2 = skin‐on red Fruit Variety 2, VEV2 = skin‐on green Fruit Variety 2, PRV2 = cortex of red fruit boiled and steamed Variety 2, PVV2 = cortex of green fruit boiled and steamed Variety 2, REBV2 = skin‐on red boiled Fruit Variety 2, VEBV2 = skin‐on green boiled Fruit Variety 2, VEVV2 = skin‐on green steamed Fruit Variety 2, REVV2 = skin‐on red steamed Fruit Variety 2, RSPBV2 = pulp of boiled red Fruit Variety 2, VSPBV2 = pulp of boiled green Fruit Variety V2, RSPVV2 = pulp of red steamed Fruit Variety 2, and VSPVV2 = pulp of steamed green Fruit Variety V2.

To determine the hypoglycemic properties of the samples, 24 initial samples were selected. To do this, a PCA was carried out on the aforementioned initial samples (active observations), with crude fiber, phenols, flavonoids, IC_50_ DPPH (2,2‐diphenyl‐1‐picrylhydrazyl), and FRAP as the active variables, as shown in Figure 2. This PCA showed a good correlation percentage (66.70%) between the factors involved. The total sample size chosen for the in vivo test is eleven (11), i.e., four (4) for variety 2 and seven (7) for variety 1. The variables fiber, FRAP, IC_50_ DPPH, and flavonoids stand out from the other variables because they are distinguished along the F1 and F2 axes. We can therefore conclude that the 11 samples cluster together in terms of these variables, hence their selection for further work. Table 1 shows the levels of correlation between the different parameters taken into account.

**TABLE 1:** Pearson’s correlation explaining the interactions between secondary metabolite composition, antioxidant properties, and crude fiber.

Variables	Phenols	Flavonoids	IC_50_ DPPH	FRAP	Crude fibers
Phenols	1	−0.230	−0.579	−0.236	−0.165
Flavonoids		1	0.435	0.179	−0.169
IC_50_ DPPH			1	0.582	−0.040
FRAP				1	0.107
Crude fibers					1

Eleven samples were selected following the PCA: 07 for the local variety (V1) and 04 for the modern variety (V2). These samples were PVV1 = cortex of green fruit boiled and steamed variety 1, REBV1 = skin‐on red boiled fruit variety 1, VEBV1 = skin‐on green boiled fruit variety 1, VEVV1 = skin‐on green steamed fruit variety 1, RSPBV1 = pulp of red boiled fruit variety 1, VSPBV1 = pulp of green boiled fruit variety 1, VSPVV1 = pulp of green steamed fruit variety 1, PVV2 = cortex of green fruit boiled and steamed variety 2, VEBV2 = skin‐on green boiled fruit variety 2, VSPBV2 = pulp of boiled green fruit variety V2, and VSPVV2 = pulp of steamed green fruit variety V2.

#### 2.10.1. In Vitro Inhibition of α‐Amylase by *S. aethiopicum* Extracts

Inhibition of α‐amylase was carried out using a procedure modified from McCue et al. [29]. For this, 250 μL of 0.02 M sodium phosphate buffer (pH 6.9) containing 0.5 mg/mL amylase solution was mixed with 250 μL of aqueous extract (1.25–10 mg/mL) in a test tube. The mixture was then pre‐incubated at 25°C for 10 min, after which 250 μL of 1% starch in 0.02 M sodium phosphate buffer (pH 6.9) was added and incubated at 25°C for 10 min. The reaction was stopped by adding 500 μL of dinitrosalicylic (DNS) acid reagent. The tubes were then incubated in boiling water for 5 min and cooled to room temperature. The reaction mixture was diluted with 5 mL of distilled water, and the absorbance was measured at 540 nm using a spectrophotometer. A control was prepared by replacing the extract with distilled water. All tests were performed in triplicate. Alpha‐amylase inhibitory activity was calculated as percentage inhibition (% inhibition):

(4) % Inhibition=Abs control−Abs extractAbs control∗100 with Abs=absorbance.

#### 2.10.2. In Vivo Evaluation of the Hypoglycemic Activity of *S. aethiopicum* Extracts Using the Oral Glucose Tolerance Test (OGTT)

This test was carried out according to the recommendations of Woumbo et al. [30], with a few adjustments. Fifty‐two (52) Wistar albino rats of both sexes (male and female), weighing between 130 and 250 g, bred in the animal house of the Department of Biochemistry at the University of Dschang, and divided into 13 groups of 4 rats per group, were used. The rats were reared for two months under standard conditions (12‐h light and 12‐h dark) with water and a normal diet provided ad libitum [31]. The extracts were administered orally to the rats at the dose of 500 mg/kg based on previous works [32] and water ad libitum during the treatment. Initial blood glucose levels were taken before the treatments. After treatment, the animals’ blood glucose levels were measured every 30 min for 2 h, and at the end, the percentage change in blood glucose compared with the initial level was calculated. A small amount of blood was taken from the rats’ tails to measure blood glucose levels. This study was carried out with due regard to the welfare of animals, with respect of the internationally accepted standard ethical guidelines for laboratory animals’ use and care prescribed by the European Union Institutional Ethics Committee on Animal Care (Council EEC 86/609/EEC of November 24, 1986), and approval was received from the Institutional Ethics Committee of the University of Douala with the ethical clearance number 2714CEI‐Udo/06/2024/M. The distribution is shown in Table 2.

**TABLE 2:** Breakdown of animals by type of treatment.

Groups	Diets
TN	2 g/kg of glucose of body weight
TNe	5 mL/kg distilled water of body weight
TTM	2 g/kg glucose + 10 mg/kg of metformin
PVV1	2 g/kg glucose + 500 mg/kg PVV1 extracts
REBV1	2 g/kg glucose + 500 mg/kg REBV1 extracts
VEBV1	2 g/kg glucose + 500 mg/kg VEBV1 extracts
VEVV1	2 g/kg glucose + 500 mg/kg VEVV1 extracts
RSPBV1	2 g/kg glucose + 500 mg/kg RSBV1 extracts
VSPBV1	2 g/kg glucose + 500 mg/kg VSPBV1 extracts
VSPVV1	2 g/kg glucose + 500 mg/kg VSPVV1 extracts
PVV2	2 g/kg glucose + 500 mg/kg PVV2 extracts
VEVV2	2 g/kg glucose + 500 mg/kg VEVV2 extracts
VSPBV2	2 g/kg glucose + 500 mg/kg VSPBV2 extracts
VSPVV2	2 g/kg glucose + 500 mg/kg VSPVV2 extracts

*Note:* TN = negative control, TNe = neutral control, TTM = metformin‐treated control, PVV1 = cortex of green fruit boiled and steamed Variety 1, REBV1 = skin‐on red boiled Fruit Variety 1, VEBV1 = skin‐on green boiled Fruit Variety 1, VEVV1 = skin‐on green steamed Fruit Variety 1, RSPBV1 = pulp of red boiled Fruit Variety 1, VSPBV1 = pulp of green boiled Fruit Variety 1, VSPVV1 = pulp of green steamed Fruit Variety 1, PVV2 = cortex of green fruit boiled and steamed Variety 2, VEBV2 = skin‐on green boiled Fruit Variety 2, VSPBV2 = pulp of boiled green Fruit Variety V2, and VSPVV2 = pulp of steamed green Fruit Variety V2.

### 2.11. Statistical Analysis

Minitab 18 software was used to analyze the results, which were expressed as mean ± standard deviation using Microsoft Office 2016. The analysis of variance (ANOVA) test at a 5% probability threshold was used to determine the difference between the means of different samples, and when a significant difference was observed, the Fisher test was applied to identify the point of difference between different variables concerning antinutrients, proximate composition, micronutrients, color, total phenol, and flavonoid content. However, the Dunnett test was used for the analysis of IC50, FRAP, and alpha‐amylase inhibition capacity. PCA was performed to determine correlations between responses using XLSTAT Version 2016 software. The samples were analyzed in triplicate.

## 3. Results and Discussion

### 3.1. Results

#### 3.1.1. Antinutrient Composition of Samples

Table 3 shows the influence of ripeness stage and culinary treatment on the content of some antinutrient compounds in the different varieties. This table shows that the concentration of phytates ranged from 80.16 to 85.20 mg/100 g in variety V1 of untreated samples, representing the highest phytate content compared to variety V2, where the values ranged from 67.12 to 74.58 mg/100 g. Regarding oxalate content, variety V2 had the highest levels, with the highest content observed in the red stage (172.28 mg/100 g). Condensed tannin content varied slightly, between 5.01 and 6.48 mg/100 g in the V1 variety and between 4.95 and 5.29 mg/100 g in the V2 variety, with the highest levels observed in the green stage of both varieties; the same was true for hydrolyzable tannins in the V1 variety (4.13–4.70 mg/100 g). In the treated samples, phytate content ranged from 75.26 to 84.74 mg/100 g in variety V1, indicating lower levels than in variety V2, where values ranged significantly from 103.74 to 127.92 mg/100 g. It should also be noted that these antinutrient contents varied depending on the treatment method applied and the ripeness stage of the samples.

**TABLE 3:** Antinutrient content of samples as a function of ripening stage and treatments.

Samples	Parameters				
	Phytates	Oxalates	Condensed tannins	Hydrolyzable tannins	Saponins
*Samples before culinary treatments*					
REV1	80.16 ± 1.67^h^	64.50 ± 2.12^d^	5.01 ± 0.18^bc^	4.130 ± 0.26^b^	0.016 ± 0.0005^d^
REV2	174.58 ± 3.46^a^	172.28 ± 1.93^a^	4.95 ± 0.16^c^	4.048 ± 0.10^b^	0.016 ± 0.0005^d^
VEV1	85.20 ± 1.39^g^	98.50 ± 0.71^c^	6.48 ± 0.14^a^	4.707 ± 0.06^a^	0.016 ± 0.0005^d^
VEV2	67.12 ± 0.09^j^	116.94 ± 2.19^b^	5.29 ± 0.16^b^	3.981 ± 0.15^b^	0.016 ± 0.0005^d^

*Pulp and whole fruits after boiling*					
REBV1	92.66 ± 1.60^f^	72.50 ± 3.74^ij^	5.61 ± 0.06^a^	3.70 ± 0.08^cd^	0.016 ± 0.0005^d^
VEBV1	80.01 ± 0.96^h^	73.37 ± 2.10^hij^	3.82 ± 0.16^de^	3.695 ± 0.04^cd^	0.016 ± 0.0005^d^
RSPBV1	84.75 ± 0.96^g^	65.00 ± 3.54^g^	3.81 ± 0.13^de^	3.457 ± 0.06^f^	0.016 ± 0.0005^d^
VSPBV1	91.53 ± 0.30^f^	84.56 ± 2.62^f^	4.27 ± 0.12^cd^	3.28 ± 0.11^ij^	0.016 ± 0.0005^d^
REBV2	113.91 ± 2.88^d^	123.33 ± 3.77^b^	3.54 ± 0.49^ef^	3.548 ± 0.06^ef^	0.016 ± 0.0005^d^
VEBV2	103.74 ± 0.08^e^	81.33 ± 1.65^fg^	4.39 ± 0.12^cd^	3.423 ± 0.06^gh^	0.016 ± 0.0005^d^
RSPBV2	125.89 ± 2.38^b^	51.39 ± 2.22^h^	3.47 ± 0.59^ef^	3.434 ± 0.07^fgh^	0.016 ± 0.0005^d^
VSPBV2	120.01 ± 2.88^c^	149.65 ± 3.11^a^	3.79 ± 0.61^def^	3.62 ± 0.10^de^	0.016 ± 0.0005^d^

*Pulp and whole fruits after steaming*					
VEVV1	91.53 ± 0.20^f^	75.32 ± 1.71^hi^	4.35 ± 0.02^cd^	3.88 ± 0.09^b^	0.016 ± 0.0005^d^
REVV1	84.75 ± 0.96^g^	65.00 ± 3.54^k^	3.07 ± 0.48^f^	3.457 ± 0.06^fgh^	0.016 ± 0.0005^d^
VSPVV1	75.26 ± 2.88^i^	56.41 ± 1.30^L^	5.39 ± 0.08^ab^	3.61 ± 0.02^de^	0.016 ± 0.0005^d^
RSPVV1	83.75 ± 0.96^g^	66.00 ± 3.54^g^	3.71 ± 0.13^de^	3.46 ± 0.06^f^	0.016 ± 0.0005^d^
VEVV2	120.01 ± 2.88^c^	54.83 ± 2.32^L^	5.26 ± 1.44^ab^	4.422 ± 0.04^a^	0.016 ± 0.0005^d^
REVV2	125.10 ± 2.88^b^	51.39 ± 2.22^L^	4.18 ± 0.14^cde^	3.434 ± 0.07^fgh^	0.016 ± 0.0005^d^
VSPVV2	127.92 ± 1.39^b^	109.00 ± 3.94^c^	4.70 ± 0.16^bc^	3.707 ± 0.08^cd^	0.016 ± 0.0005^d^
RSPVV2	121.01 ± 2.88^c^	150.64 ± 3.11^a^	3.80 ± 0.51^def^	3.61 ± 0.10^de^	0.016 ± 0.0005^d^

*Cortex of boiled and steamed fruits*					
PRV1	60.34 ± 0.96^k^	91.50 ± 2.12^e^	4.04 ± 0.12^cde^	3.388 ± 0.06^hi^	0.53 ± 0.13^a^
PVV1	80.68 ± 1.92^h^	68.76 ± 1.31^ij^	3.95 ± 0.04^de^	3.515 ± 0.11^efg^	0.44 ± 0.13^b^
PRV2	77.07 ± 3.05^i^	102.17 ± 1.25^d^	4.18 ± 0.13^cde^	3.207 ± 0.08^j^	0.44 ± 0.23^b^
PVV2	91.53 ± 0.10^f^	74.00 ± 3.54^hi^	3.96 ± 0.23^de^	3.411 ± 0.05^ghi^	0.36 ± 0.16^c^

*Note:* Values with different letters in the same column are significantly different (*p* < 0.05), REV1 = skin‐on red Fruit Variety 1, VEV1 = skin‐on green Fruit Variety V1, PRV1 = cortex of red fruit boiled and steamed Variety 1, PVV1 = cortex of green fruit boiled and steamed Variety 1, REBV1 = skin‐on red boiled Fruit Variety 1, VEBV1 = skin‐on green boiled Fruit Variety 1, VEVV1 = skin‐on green steamed Fruit Variety 1, REVV1 = skin‐on red steamed Fruit Variety 1, RSPBV1 = pulp of red boiled Fruit Variety 1, VSPBV1 = pulp of green boiled Fruit Variety 1, VSPVV1 = pulp of green steamed Fruit Variety 1, RSPVV1 = pulp of red steamed fruit variety, REV2 = skin‐on red Fruit Variety 2, VEV2 = skin‐on green Fruit Variety 2, PRV2 = cortex of red fruit boiled and steamed Variety 2, PVV2 = cortex of green fruit boiled and steamed Variety 2, REBV2 = skin‐on red boiled Fruit Variety 2, VEBV2 = skin‐on green boiled Fruit Variety 2, VEVV2 = skin‐on green steamed Fruit Variety 2, REVV2 = skin‐on red steamed Fruit Variety 2, RSPBV2 = pulp of boiled red Fruit Variety 2, VSPBV2 = pulp of boiled green Fruit Variety V2, RSPVV2 = pulp of red steamed Fruit Variety 2, and VSPVV2 = pulp of steamed green Fruit Variety V2.

#### 3.1.2. Extraction Yield, Total Phenol, and Flavonoid Content of Samples

Table 4 shows the extraction yield, the total phenol, and flavonoid contents of the aqueous extracts of *S. aethiopicum* powders. This table shows that the skin‐on green boiled fruit variety 1 (VEBV1) and the skin‐on red boiled fruit variety 2 (REBV2) of *S. aethiopicum* extract gave the highest yield with the values 13.07% and 18.01%, respectively. The lowest yield was 1.58% for V1 and 5.14% for V2, respectively, in the pulp of green steamed fruit variety 1 (VSPVV1) and in the skin‐on red boiled fruit variety 2 (REBV2) extracts. Concerning total phenol and flavonoid content, there was a significant difference (*p* < 0.05) between the two varieties. Total phenol content was higher in the red samples of both varieties (101.07 ± 1.04 and 101.07 ± 1.04 mg GAE/g) before treatment compared to the green samples of both varieties (78.07 ± 0.90 and 81.50 ± 2.83 mg GAE/g). In the treated samples, the variation in total phenol content ranged from 45.27 ± 2.01 to 95.73 ± 0.82 mg GAE/g in variety V1 and from 42.61 ± 2.81 to 70.42 ± 2.02 mg GAE/g in variety V2. Regarding flavonoid content, the highest value was 12.64 ± 0.06 mg EC/g extract for the pulp of boiled green fruit of variety V1, while the lowest value was 9.10 ± 0.19 mg EC/g extract for the cortex of boiled and steamed red fruit of variety V1.

**TABLE 4:** Extraction yield, total phenol, and flavonoid content of different *S. aethiopicum* samples.

Samples	Parameters		
	Yield (%)	Total phenols (mg EAG/g extract)	Flavonoids (mg EC/g extract)
*Samples before culinary treatments*			
REV1	11.01 ± 0.27^a^	101.07 ± 1.04^a^	9.72 ± 0.12^b^
REV2	17.31 ± 0.18^a^	101.01 ± 1.04^a^	10.23 ± 0.16^ab^
VEV1	3.20 ± 0.05^b^	78.07 ± 0.90^c^	10.23 ± 0.27^ab^
VEV2	13.06 ± 2.29^b^	81.50 ± 2.83^c^	10.23 ± 0.38^ab^

*Pulp and whole fruits after boiling*			
REBV1	3.24 ± 0.11^b^	45.80 ± 0.92^g^	10.78 ± 0.06^ab^
VEBV1	13.07 ± 0.81^a^	40.21 ± 3.69^g^	10.81 ± 0.45^ab^
RSPBV1	4.63 ± 0.92^b^	62.05 ± 3.23^e^	10.41 ± 0.19^ab^
VSPBV1	3.29 ± 0.75^b^	45.27 ± 2.01^g^	12.64 ± 0.06^a^
REBV2	5.14 ± 0.21^b^	42.61 ± 2.81^g^	10.34 ± 0.22^ab^
VEBV2	6.11 ± 0.52^b^	56.86 ± 0.59^f^	10.30 ± 0.19^ab^
RSPBV2	10.22 ± 0.45^b^	59.94 ± 0.75^e^	9.39 ± 0.12^b^
VSPBV2	8.23 ± 0.41^b^	48.54 ± 0.46^g^	10.05 ± 0.38^ab^

*Pulp and whole fruits after steaming*			
VEVV1	4.46 ± 0.48^b^	48.47 ± 4.88^g^	12.62 ± 0.56^a^
REVV1	1.90 ± 0.17^b^	95.73 ± 0.82^b^	11.33 ± 0.69^a^
VSPVV1	1.58 ± 0.25^c^	48.46 ± 1.22^g^	12.02 ± 0.31^a^
RSPVV1	5.55 ± 1.65^b^	61.05 ± 3.13^e^	10.51 ± 0.23^ab^
VEVV2	6.74 ± 0.48^b^	59.63 ± 2.38^e^	11.00 ± 0.66^a^
REVV2	18.01 ± 4.04^a^	70.42 ± 2.02^d^	9.46 ± 0.31^b^
VSPVV2	12.00 ± 1.22^b^	61.07 ± 2.52^e^	10.08 ± 0.58^ab^
RSPVV2	10.44 ± 0.34^b^	48.50 ± 0.56^g^	10.05 ± 0.38^ab^

*Cortex of boiled and steamed fruits*			
PRV1	2.50 ± 0.31^b^	83.53 ± 1.73^c^	9.10 ± 0.19^b^
PVV1	3.52 ± 1.43^b^	36.57 ± 0.86^h^	10.23 ± 0.27^ab^
PRV2	8.50 ± 2.43^b^	84.02 ± 1.81^c^	10.92 ± 0.38^ab^
PVV2	7.84 ± 0.82^b^	47.40 ± 2.44^g^	10.08 ± 0.29^ab^

*Note:* Values with different letters in the same column are significantly different (*p* < 0.05), REV1 = skin‐on red Fruit Variety 1, VEV1 = skin‐on green Fruit Variety V1, PRV1 = cortex of red fruit boiled and steamed Variety 1, PVV1 = cortex of green fruit boiled and steamed Variety 1, REBV1 = skin‐on red boiled Fruit Variety 1, VEBV1 = skin‐on green boiled Fruit Variety 1, VEVV1 = skin‐on green steamed Fruit Variety 1, REVV1 = skin‐on red steamed Fruit Variety 1, RSPBV1 = pulp of red boiled Fruit Variety 1, VSPBV1 = pulp of green boiled Fruit Variety 1, VSPVV1 = pulp of green steamed Fruit Variety 1, RSPVV1 = pulp of red steamed fruit variety, REV2 = skin‐on red Fruit Variety 2, VEV2 = skin‐on green Fruit Variety 2, PRV2 = cortex of red fruit boiled and steamed Variety 2, PVV2 = cortex of green fruit boiled and steamed Variety 2, REBV2 = skin‐on red boiled Fruit Variety 2, VEBV2 = skin‐on green boiled Fruit Variety 2, VEVV2 = skin‐on green steamed Fruit Variety 2, REVV2 = skin‐on red steamed Fruit Variety 2, RSPBV2 = pulp of boiled red Fruit Variety 2, VSPBV2 = pulp of boiled green Fruit Variety V2, RSPVV2 = pulp of red steamed Fruit Variety 2, and VSPVV2 = pulp of steamed green Fruit Variety V2.

#### 3.1.3. Proximate Composition of Samples

Table 5 shows the moisture, dry matter, ash, organic matter, and fiber contents of various samples. It appears that fiber content varied between 14.29 ± 0.04 and 30.82 ± 0.10%DM for variety V1 and between 15.14 ± 0.02 and 29.39 ± 0.02%DM for variety V2. The highest fiber contents were also found in the individual pulps (26.76 ± 0.16 and 30.82 ± 0.10%DM for V1, and 34.34 ± 0.04 and 43.34 ± 0.12%DM for V2). The untreated samples had the lowest fiber content. Organic matter percentages were also recorded, varying between 91.27 ± 0.21 and 96.93 ± 0.02% for variety V1 and between 89.81 ± 0.31 and 96.41 ± 0.11% for variety V2.

**TABLE 5:** Moisture, ash, organic matter, and fiber contents of different samples.

Samples	Dry matter (DM) (%)	Moisture	Ash (%DM)	Organic matter (%DM)	Crude fiber (%DM)
*Samples before culinary treatments*					
REV1	90.25 ± 0.18^e^	9.75 ± 0.18^a^	7.69 ± 0.06^b^	92.30 ± 0.06^e^	14.29 ± 0.04^g^
REV2	91.98 ± 0.11^c^	8.02 ± 0.11^c^	7.10 ± 0.02^c^	92.90 ± 0.02^d^	15.14 ± 0.02^f^
VEV1	91.10 ± 0.07^d^	8.89 ± 0.07^b^	8.73 ± 0.21^a^	91.27 ± 0.21^f^	15.19 ± 0.04^f^
VEV2	93.66 ± 0.27^a^	6.33 ± 0.27^e^	6.95 ± 0.14^c^	93.04 ± 0.14^d^	18.73 ± 0.05^e^

*Pulp and whole fruits after boiling*					
REBV1	88.32 ± 0.42^f^	11.68 ± 0.42^cd^	5.94 ± 0.09^f^	94.05 ± 0.09^g^	21.16 ± 0.09^e^
VEBV1	88.99 ± 0.07^c^	11.01 ± 0.07^e^	3.90 ± 0.08^i^	96.09 ± 0.30^a^	15.59 ± 0.01^k^
RSPBV1	85.99 ± 0.08^de^	14.01 ± 0.08^a^	4.82 ± 0.10^h^	95.17 ± 0.10^d^	17.95 ± 0.01^g^
VSPBV1	88.70 ± 0.01^g^	11.30 ± 0.01^de^	5.62 ± 0.10^g^	94.37 ± 0.27^b^	17.67 ± 0.18^h^
REBV2	90.16 ± 0.19^e^	9.84 ± 0.19^f^	7.17 ± 0.22^c^	92.83 ± 0.22^de^	26.85 ± 0.05^c^
VEBV2	89.11 ± 0.18^c^	10.89 ± 0.18^e^	8.11 ± 0.16^b^	91.88 ± 0.16^h^	28.06 ± 0.06^b^
RSPBV2	90.41 ± 0.36^b^	9.58 ± 0.36^f^	6.28 ± 0.02^e^	93.71 ± 0.02^g^	15.87 ± 0.06^j^
VSPBV2	91.38 ± 0.22^b^	8.62 ± 0.22^g^	6.79 ± 0.17^d^	93.20 ± 0.17^e^	18.85 ± 0.04^f^

*Pulp and whole fruits after steaming*					
VEVV1	87.54 ± 0.15^f^	12.46 ± 0.15^b^	4.10 ± 0.12^i^	95.89 ± 0.12^a^	14.91 ± 0.02^l^
REVV1	88.68 ± 0.26^cde^	11.32 ± 0.26^cde^	7.04 ± 0.03^cd^	92.95 ± 0.03^fg^	21.16 ± 0.06^e^
VSPVV1	87.46 ± 0.26^cd^	12.53 ± 0.26^b^	7.31 ± 0.21^c^	92.69 ± 0.21^c^	17.35 ± 0.04^i^
RSPVV1	86.10 ± 0.08^de^	14.50 ± 0.08^a^	5.32 ± 0.10^h^	94.17 ± 0.20^d^	17.66 ± 0.02^g^
VEVV2	91.77 ± 0.10^a^	8.22 ± 0.10^g^	10.19 ± 0.31^a^	89.81 ± 0.31^i^	29.39 ± 0.02^a^
REVV2	90.25 ± 0.24^b^	9.75 ± 0.24^f^	6.20 ± 0.08^ef^	93.80 ± 0.08^de^	25.99 ± 0.07^d^
VSPVV2	88.19 ± 0.28^a^	11.81 ± 0.28^c^	6.25 ± 0.01^ef^	93.75 ± 0.01^f^	15.92 ± 0.04^j^
RSPVV2	91.41 ± 0.56^b^	10.58 ± 0.46^f^	6.30 ± 0.06^e^	94.51 ± 0.05^g^	16.80 ± 0.07^j^

*Cortex of boiled and steamed fruits*					
PRV1	91.19 ± 0.10^d^	8.80 ± 0.10^b^	3.87 ± 0.14^d^	96.12 ± 0.14^c^	26.76 ± 0.16^d^
PVV1	91.31 ± 0.06^d^	8.68 ± 0.06^b^	3.06 ± 0.02^f^	96.93 ± 0.02^a^	30.82 ± 0.10^c^
PRV2	92.76 ± 0.14^b^	7.24 ± 0.14^d^	3.95 ± 0.12^d^	96.04 ± 0.12^c^	34.34 ± 0.04^b^
PVV2	93.50 ± 0.26^a^	6.50 ± 0.26^e^	3.59 ± 0.11^e^	96.41 ± 0.11^b^	43.34 ± 0.12^a^

*Note:* Values with different letters in the same column are significantly different (*p* < 0.05), REV1 = skin‐on red Fruit Variety 1, VEV1 = skin‐on green Fruit Variety V1, PRV1 = cortex of red fruit boiled and steamed Variety 1, PVV1 = cortex of green fruit boiled and steamed Variety 1, REBV1 = skin‐on red boiled Fruit Variety 1, VEBV1 = skin‐on green boiled Fruit Variety 1, VEVV1 = skin‐on green steamed Fruit Variety 1, REVV1 = skin‐on red steamed Fruit Variety 1, RSPBV1 = pulp of red boiled Fruit Variety 1, VSPBV1 = pulp of green boiled Fruit Variety 1, VSPVV1 = pulp of green steamed Fruit Variety 1, RSPVV1 = pulp of red steamed fruit variety, REV2 = skin‐on red Fruit Variety 2, VEV2 = skin‐on green Fruit Variety 2, PRV2 = cortex of red fruit boiled and steamed Variety 2, PVV2 = cortex of green fruit boiled and steamed Variety 2, REBV2 = skin‐on red boiled Fruit Variety 2, VEBV2 = skin‐on green boiled Fruit Variety 2, VEVV2 = skin‐on green steamed Fruit Variety 2, REVV2 = skin‐on red steamed Fruit Variety 2, RSPBV2 = pulp of boiled red Fruit Variety 2, VSPBV2 = pulp of boiled green Fruit Variety V2, RSPVV2 = pulp of red steamed Fruit Variety 2, and VSPVV2 = pulp of steamed green Fruit Variety V2.

#### 3.1.4. In Vitro Antioxidant Properties of Aqueous *S. aethiopicum* Extracts

**FIGURE 3:**
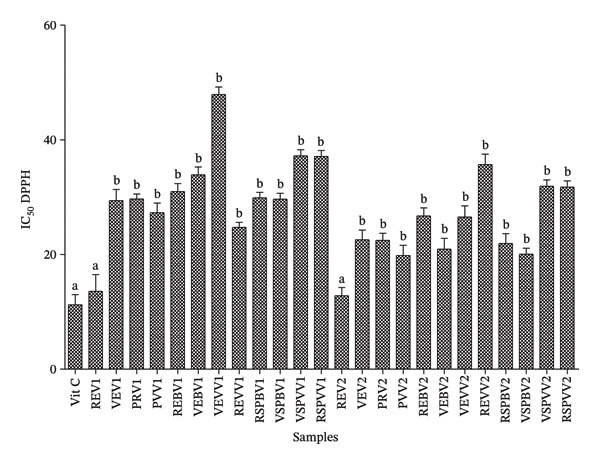
IC_50_ for the 2,2‐diphenyl‐1‐picrylhydrazyl (DPPH) scavenging activity of extracts from different samples. Vit C = vitamin C, IC = inhibitory concentration, REV1 = skin‐on red Fruit Variety 1, VEV1 = skin‐on green Fruit Variety V1, PRV1 = cortex of red fruit boiled and steamed Variety 1, PVV1 = cortex of green fruit boiled and steamed Variety 1, REBV1 = skin‐on red boiled Fruit Variety 1, VEBV1 = skin‐on green boiled Fruit Variety 1, VEVV1 = skin‐on green steamed Fruit Variety 1, REVV1 = skin‐on red steamed Fruit Variety 1, RSPBV1 = pulp of red boiled Fruit Variety 1, VSPBV1 = pulp of green boiled Fruit Variety 1, VSPVV1 = pulp of green steamed Fruit Variety 1, RSPVV1 = pulp of red steamed fruit variety, REV2 = skin‐on red Fruit Variety 2, VEV2 = skin‐on green Fruit Variety 2, PRV2 = cortex of red fruit boiled and steamed Variety 2, PVV2 = cortex of green fruit boiled and steamed Variety 2, REBV2 = skin‐on red boiled Fruit Variety 2, VEBV2 = skin‐on green boiled Fruit Variety 2, VEVV2 = skin‐on green steamed Fruit Variety 2, REVV2 = skin‐on red steamed Fruit Variety 2, RSPBV2 = pulp of boiled red Fruit Variety 2, VSPBV2 = pulp of boiled green Fruit Variety V2, RSPVV2 = pulp of red steamed Fruit Variety 2, and VSPVV2 = pulp of steamed green Fruit Variety V2.

Figure 3 shows the IC_50_ DPPH activity of extracts from different samples. The results revealed that there was no significant difference between skin‐on red samples (samples REV1 and REV2) and vitamin C with the respective values of 13.58, 12.79, and 11.22 μg/mL. Apart from these skin‐on red samples, it can be seen that the treatments significantly increased the IC_50_ DPPH values of the other samples compared to that of vitamin C. The highest IC_50_ DPPH value was found in the sample with skin‐on green steamed fruit from the local variety (VEVV1 = 47.89 μg/mL).

**FIGURE 4:**
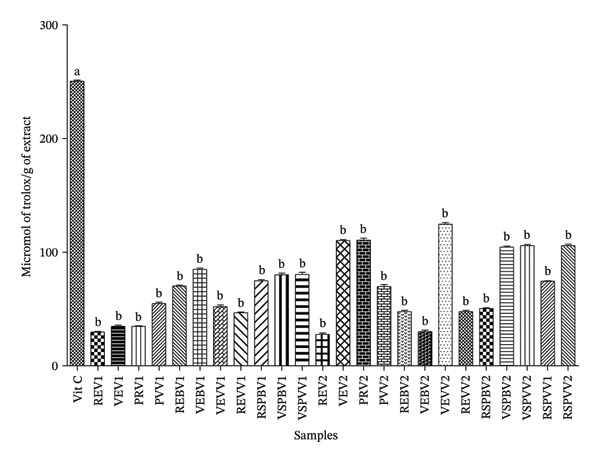
Concentration in μmol of trolox/g of extract on the ferric reducing power in samples (FRAP test). Vit C = vitamin C, REV1 = skin‐on red Fruit Variety 1, VEV1 = skin‐on green Fruit Variety V1, PRV1 = cortex of red fruit boiled and steamed Variety 1, PVV1 = cortex of green fruit boiled and steamed Variety 1, REBV1 = skin‐on red boiled Fruit Variety 1, VEBV1 = skin‐on green boiled Fruit Variety 1, VEVV1 = skin‐on green steamed Fruit Variety 1, REVV1 = skin‐on red steamed Fruit Variety 1, RSPBV1 = pulp of red boiled Fruit Variety 1, VSPBV1 = pulp of green boiled Fruit Variety 1, VSPVV1 = pulp of green steamed Fruit Variety 1, RSPVV1 = pulp of red steamed fruit variety, REV2 = skin‐on red Fruit Variety 2, VEV2 = skin‐on green Fruit Variety 2, PRV2 = cortex of red fruit boiled and steamed Variety 2, PVV2 = cortex of green fruit boiled and steamed Variety 2, REBV2 = skin‐on red boiled Fruit Variety 2, VEBV2 = skin‐on green boiled Fruit Variety 2, VEVV2 = skin‐on green steamed Fruit Variety 2, REVV2 = skin‐on red steamed Fruit Variety 2, RSPBV2 = pulp of boiled red Fruit Variety 2, VSPBV2 = pulp of boiled green Fruit Variety V2, RSPVV2 = pulp of red steamed Fruit Variety 2, and VSPVV2 = pulp of steamed green Fruit Variety V2.

The ability of extracts of different powders to reduce ferric ion (FRAP test) is shown in Figure 4. This figure revealed that regardless of the treatment and ripeness state of fruits, the FRAP value of vitamin C was significantly very high (250.33 μmol trolox/g extract). No samples of the local variety had a FRAP value above 100 μmol trolox/g extract. However, only a few samples of the modern variety had FRAP values above this reference, such as VEV2, PRV2, VEVV2, VSPBV2, and VSPVV2, whose values were, respectively, 110, 110, 124, 104, and 106 μmol trolox/g extract.

#### 3.1.5. Coloring of Different *S. aethiopicum* Powders From Selected Samples

Table 6 shows the mean values of the Cartesian color coordinates for the 11 samples, while Table 7 lists the polar coordinates of interest derived from them. Essentially, samples VSPBV2 (*L* ^∗^ = 51.57), VEBV1 (*L* ^∗^ = 50.26), VSPBV1 (*L* ^∗^ = 49.95), and PVV1 (*L* ^∗^ = 48.45) exhibited the best luminosities and were therefore considered to be ≥ 50 on a traditional 0–100 scale. In terms of chromaticity, RSPBV1 (*C* ^∗^ = 25.73) and VSPBV2 (*C* ^∗^ = 25.25) showed the highest color intensities, with REBV1 (*C* ^∗^ = 6.18) and VEBV1 (*C* ^∗^ = 5.57) at the bottom of the list. For the colorimetric hue, the values for PVV2 (*h* ^∗^ = 83.72), VEVV1 (*h* ^∗^ = 81.82°), and VSPBV2 (*h* ^∗^ = 80.65) were almost similar and highest at around 90°, unlike the hues noted for the other samples, which gradually move away from the right angle at around 0°.

**TABLE 6:** *L* ^∗^ *a* ^∗^ *b* ^∗^ color parameters of selected samples.

Cartesian color coordinates				
Samples	*L* ^∗^	*a* ^∗^	*b* ^∗^	WI
PVV1	48.45 ± 1.77^bcd^	5.57 ± 0.21^ef^	18.86 ± 0.54^ef^	44.27 ± 1.46^bc^
REBV1	46.64 ± 0.99^de^	10.83 ± 1.03^b^	21.73 ± 0.60^bcd^	41.53 ± 0.24^d^
VEBV1	50.26 ± 0.38^ab^	4.43 ± 0.014^f^	22.35 ± 0.07^ab^	45.29 ± 0.03^ab^
VEVV1	46.20 ± 0.21^e^	2.97 ± 0.05^g^	20.66 ± 2.33^bcde^	42.27 ± 0.82^d^
RSPBV1	47.89 ± 0.56^cde^	10.24 ± 0.13^bc^	23.61 ± 0.20^a^	41.87 ± 0.40^d^
VSPBV1	49.95 ± 0.49^ab^	4.34 ± 0.27^fg^	20.11 ± 0.04^def^	45.88 ± 0.49^a^
VSPVV1	47.07 ± 1.15^de^	6.18 ± 0.57^e^	20.66 ± 0.65^bcde^	42.84 ± 0.77^cd^
PVV2	49.25 ± 0.72^d^	1.93 ± 0.20^g^	17.55 ± 0.47^cd^	46.26 ± 0.51^bc^
VEVV2	47.20 ± 0.12^e^	3.63 ± 0.07^f^	16.58 ± 0.13^d^	44.54 ± 0.15^d^
VSPBV2	51.57 ± 0.50^bc^	4.10 ± 0.85^f^	24.91 ± 6.05^a^	45.24 ± 2.24^cd^
VSPVV2	46.57 ± 0.60^e^	3.71 ± 0.60^f^	16.83 ± 1.06^d^	43.85 ± 0.21^d^

*Note:* Values with different letters in the same column are significantly different (*p* < 0.05). PVV1 = cortex of green fruit boiled and steamed Variety 1, REBV1 = skin‐on red boiled Fruit Variety 1, VEBV1 = skin‐on green boiled Fruit Variety 1, VEVV1 = skin‐on green steamed Fruit Variety 1, RSPBV1 = pulp of red boiled Fruit Variety 1, VSPBV1 = pulp of green boiled Fruit Variety 1, VSPVV1 = pulp of green steamed Fruit Variety 1 PVV2 = cortex of green fruit boiled and steamed Variety 2, VEVV2 = skin‐on green steamed Fruit Variety 2, VSPBV2 = pulp of boiled green Fruit Variety V2, and VSPVV2 = pulp of steamed green Fruit Variety V2.

Abbreviation: WI = Whiteness Index.

**TABLE 7:** *L* ^∗^ *C* ^∗^ *h* ^∗^ parameters resulting from the average *L* ^∗^ *a* ^∗^ *b* ^∗^ strings of selected samples.

Polar coordinates for color parameters			
Samples	*L* ^∗^	*C* ^∗^	*h* ^∗^
PVV1	48.45	19.67	73.55
REBV1	46.64	24.28	63.51
VEBV1	50.26	22.78	78.79
VEVV1	46.20	20.87	81.82
RSPBV1	47.89	25.73	66.55
VSPBV1	49.95	20.57	77.82
VSPVV1	47.07	21.56	73.35
PVV2	49.25	17.66	83.72
VEVV2	47.20	16.97	77.65
VSPBV2	51.50	25.25	80.65
VSPVV2	46.50	17.23	77.57

*Note:* *L* ^∗^ = brightness, *C* ^∗^: chromaticity, *h* ^∗^ = hue, PVV1 = cortex of green fruit boiled and steamed Variety 1, REBV1 = skin‐on red boiled Fruit Variety 1, VEBV1 = skin‐on green boiled Fruit Variety 1, VEVV1 = skin‐on green steamed Fruit Variety 1, RSPBV1 = pulp of red boiled Fruit Variety 1, VSPBV1 = pulp of green boiled Fruit Variety 1, VSPVV1 = pulp of green steamed Fruit Variety 1, PVV2 = cortex of green fruit boiled and steamed Variety 2, VEVV2 = skin‐on green steamed Fruit Variety 2, VSPBV2 = pulp of boiled green Fruit Variety V2, and VSPVV2 = pulp of steamed green Fruit Variety V2.

#### 3.1.6. Bioactive Compounds Contained in *S. aethiopicum* Varieties

**FIGURE 5:**
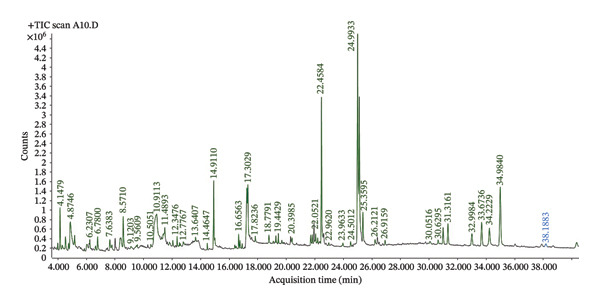
Spectrum of bioactive compounds contained in Variety V1 of *Solanum aethiopicum* L. fruits.

**FIGURE 6:**
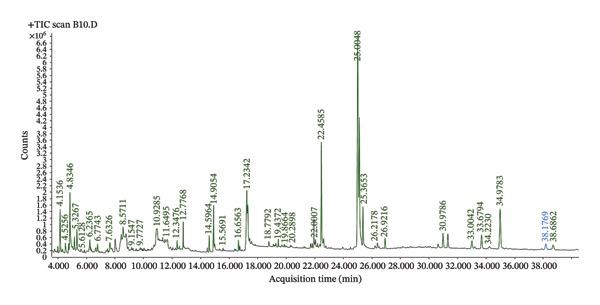
Spectrum of bioactive compounds contained in Variety V2 of *Solanum aethiopicum* L. fruits.

Identification of the bioactive compounds in the two varieties of *S. aethiopicum* revealed that 73 compounds (Figure 5), including 29 major compounds, are present in variety V1 (Table 8), and 71 compounds (Figure 6), including 24 major compounds, are present in variety V2 (Table 9). A comparison of the compounds in the two varieties shows that they share 13 similar bioactive compounds. Figures 5 and 6 are used to prepare Tables 8 and 9, respectively.

**TABLE 8:** Bioactive compounds contained in samples of *S. aethiopicum* of Variety V1.

N°	RT (min)	Compound name	Formula	Library molecular weight	Area (%)
1	4.1479	4H‐Pyran‐4‐one, 2,3‐dihydro‐3,5‐dihydroxy‐6‐methyl	C_6_H_8_O_4_	144	1.94
2	4.8746	Catechol	C_6_H_6_0_2_	110	4.00
3	6.7800	Ethanone, 1‐(2‐hydroxy‐5‐methylphenyl)	C_9_H_10_O_2_	150.1	0.91
4	7.6383	DL‐Proline, 5‐oxo‐, methyl ester	C_6_H_9_NO_3_	143.1	0.83
5	8.5710	4‐Vinylbenzene‐1,2‐diol	C_8_H_8_O_2_	136.1	1.77
6	9.5609	2,4‐Di‐tert‐butylphenol	C_14_H_22_O	206.2	0.12
7	10.9113	1,2,3,5‐Cyclohexanetetrol (1.alpha.,2.beta.,5.beta.)	C_6_H_12_O_4_	148.1	7.60
8	12.3476	(E)‐4‐(3‐Hydroxyprop‐1‐yl)‐2‐methoxyphenol	C_10_H_12_O_3_	180.1	0.41
9	13.6407	5‐Isopropenyloxymethylene‐3,3‐dimethylcyclohexanone	C_12_H_18_O_2_	194.1	0.23
10	14.4647	Hexadecanoic acid and methyl ester	C_17_H_34_O_2_	270.3	0.21
11	14.9110	n‐Hexadecanoic acid	C_16_H_32_O_2_	256.2	3.46
12	16.6563	9,12‐Octadecadienoic acid (Z,Z)‐, methyl ester	C_19_H_34_O_2_	294.3	0.58
13	17.3029	9,12,15‐Octadecatrienoid acid, (Z,Z,Z)‐	C_18_H_30_O_2_	278.2	3.45
14	18.7791	.beta.‐d‐Mannofuranoside, O‐geranyl	C_16_H_28_O_6_	316.2	0.49
15	19.4429	Glycidyl palmitate	C_19_H_36_O_3_	312.3	0.44
16	20.3985	Arachidonoyl amide	C_20_H_33_NO	303.3	0.58
17	22.0521	O‐Arachidonoylglycidol	C_23_H_36_O_3_	360.3	0.59
18	22.4584	Hexadecanoic acid, 2‐hydroxy‐1‐(hydroxymethyl)ethyl ester	C_19_H_38_O_4_	330.3	8.29
19	22.9620	Erucic acid	C_22_H_42_O_2_	338.3	0.86
20	24.5012	Cinnamoylechinadiol, TMS	C_27_H_40_O_4_Si	456.3	0.28
21	24.9933	9,12‐Octadecanoic acid (Z,Z)‐, 2,3‐dihygroxypropyl ester	C_2_1H_38_O_4_	354.3	19.09
22	26.2121	13‐Docosenamide, (Z)‐	C_22_H_43_NO	337.3	0.33
23	26.9159	2,6,10,14,18‐Pentamethyl‐2,6,10,14,18‐eicosapentaene	C_25_H_42_	342.3	0.24
24	30.6295	Stigmasta‐3,5‐diene	C_29_H_48_	396.4	0.35
25	31.3161	Vitamin E	C_29_H_50_O_2_	430.4	1.29
26	32.9984	Campesterol	C_28_H_48_O	400.4	1.15
27	34.6736	Stigmasterol	C_29_H_48_O	412.4	2.24
28	34.9840	.gamma.‐Sitosterol	C_29_H_50_O	414.4	6.65
29	38.1883	Citrost‐7‐en‐3‐ol	C_30_H_52_O	428.4	0.40

**TABLE 9:** Bioactive compounds contained in samples of *S. aethiopicum* of Variety V2.

N°	TR (min)	Compound name	Formula	Library molecular weight	Area (%)
1	4.1536	4H‐Pyran‐4‐one, 2,3‐dihydro‐3,5‐dihydroxy‐6‐methyl	C_6_H_8_O_4_	144	2.35
2	4.8346	Catechol	C_6_H_6_0_2_	110	5.06
3	6.2365	1,2,3‐Butanetriol	C_4_H_10_O_3_	106.1	0.87
4	7.6326	2‐Nonanol, acetate	C_11_H_22_O_2_	186.2	1.05
5	8.5711	4‐Vinylbenzene‐1,2‐diol	C_8_H_8_O_2_	136.1	0.66
6	9.7727	2,7‐Anhydro‐l‐galacto‐heptulofuranose	C_7_H_12_O_6_	192.1	0.25
7	10.9285	n‐Butyric acid 2‐ethylhexyl ester	C_12_H_24_O_2_	200.2	2.36
8	12.7768	1‐(2‐Hydroxyphenyl)‐2‐pyrrolidinone	C_10_H_11_NO_2_	177.1	1.23
9	14.9054	n‐Hexadecanoic acid	C_16_H_32_O_2_	256.2	2.60
10	15.5691	Propanone, 3‐(3‐methyl‐2‐benzothienyl)‐1‐phenylthio‐	C_18_H_140_S_2_	310	0.11
11	17.2342	9,12‐Octadecadienoic acid (Z,Z)‐	C_18_H_32_O_2_	280.2	5.42
12	18.7792	.beta.‐d‐Mannofuranoside, O‐geranyl	C_16_H_28_O_6_	316.2	0.33
13	19.4372	Glycidyl palmitate	C_19_H_36_O_3_	312.3	0.40
14	20.2892	Oleic acid	C_18_H_34_O_2_	282.3	0.14
15	22.0007	9‐Octadecenoic acid (Z)‐, oxiranylmethyl ester	C_21_H_38_O_3_	338.3	0.26
16	22.4585	Hexadecanoic acid, 2‐hydroxy‐1‐(hydroxymethyl)ethyl ester	C_19_H_38_O_4_	330.3	6.90
17	25.0048	9,12‐Octadecanoic acid (Z,Z)‐, 2,3‐dihygroxypropyl ester	C_21_H_38_O_4_	354.3	22.77
18	26.2178	13‐Decosemide, (Z)‐	C_22_H_43_NO	337.3	0.28
19	26.9216	Squalene	C_30_H_50_	410.4	0.63
20	30.9786	Cholesterol	C_27_H_46_O	386.4	1.22
21	33.0042	Campesterol	C_28_H_48_O	400.4	0.84
22	33.6794	Stigmasterol	C_29_H_48_O	412.4	1.53
23	34.9783	.gamma.‐Sitosterol	C_29_H_50_O	414.4	5.70
24	38.1769	Citrost‐7‐en‐3‐ol	C_30_H_52_O	428.4	0.84

#### 3.1.7. Proximate Chemical Composition, Mineral, and Reducing Sugar Content of Selected Samples

Table 10 shows the macronutrient contents of the sample powders. A significant decrease (*p* < 0.05) in the protein content of the samples cooked in boiling water (10.98 and 10.81 mg/100 g) was observed. No significant difference in lipid content was observed between the samples cooked in boiling water (5.39 mg/100 g). However, there was a significant difference (*p* < 0.05) between the steamed samples without pulp (5.58 and 4.75 mg/100 g). Additionally, the highest carbohydrate content (53.30 mg/100 g) was observed in the V1 steamed samples, while the lowest content (35.60 mg/100 g) was recorded in the V2 steamed samples.

**TABLE 10:** Macronutrient composition of selected samples of two varieties of *S. aethiopicum*.

Samples	Parameters			
	Proteins	Lipids	Carbohydrates	Reducing sugar (mg/100 g)
PVV1	ND	ND	ND	ND
REBV1	10.98 ± 0.01^e^	5.39 ± 0.01^f^	44.85 ± 0.01^f^	0.69 ± 0.03^cd^
VEBV1	10.81 ± 0.02^f^	5.39 ± 0.01^f^	53.3 ± 0.01^a^	1.02 ± 0.05^a^
VEVV1	11.49 ± 0.04^c^	4.07 ± 0.01^e^	52.97 ± 0.03^b^	0.80 ± 0.06^bc^
RSPBV1	11.25 ± 0.01^d^	4.955 ± 0.01^g^	47.01 ± 0.01^e^	0.54 ± 0.10^e^
VSPBV1	9.4 ± 0.04^g^	5.585 ± 0.02^h^	50.42 ± 0.01^d^	0.84 ± 0.12^b^
VSPVV1	14.4 ± 0.01^a^	4.745 ± 0.01^d^	43.66 ± 0.02^g^	0.27 ± 0.05^f^
PVV2	ND	ND	ND	ND
VEVV2	12.48 ± 0.04^b^	4.12 ± 0.01^c^	35.6 ± 0.01^i^	0.26 ± 0.01^f^
VSPBV2	10.81 ± 0.06^f^	2.89 ± 0.01^b^	52.04 ± 0.02^c^	1.07 ± 0.10^a^
VSPVV2	11.29 ± 0.04^d^	11.24 ± 0.01^a^	43.49 ± 0.01^h^	0.20 ± 0.04^f^

*Note:* Values with different letters in the same column are significantly different (*p* < 0.05). PVV1 = cortex of green fruit boiled and steamed Variety 1, REBV1 = skin‐on red boiled Fruit Variety 1, VEBV1 = skin‐on green boiled Fruit Variety 1, VEVV1 = skin‐on green steamed Fruit Variety 1, RSPBV1 = pulp of red boiled Fruit Variety 1, VSPBV1 = pulp of green boiled Fruit Variety 1, VSPVV1 = pulp of green steamed Fruit Variety 1 PVV2 = cortex of green fruit boiled and steamed Variety 2, VEVV2 = skin‐on green steamed Fruit Variety 2, VSPBV2 = pulp of boiled green Fruit Variety V2, and VSPVV2 = pulp of steamed green Fruit Variety V2.

Abbreviation: ND = not determined.

Table 11 shows the composition of some minerals in the selected samples. The phosphorus content ranged between 77.07 ± 0.01 and 697.39 mg/100 g in the V1 variety. The lowest content was recorded in the VEBV1 and RSPBV1 samples (68.26 and 77.07 mg/100 g, respectively). High phosphorus content was also recorded in the V2 variety, particularly in the VSPBV2 and VEV2 samples (561.10 and 376.36 mg/100 g, respectively). There was also significant variation in the Ca, Mg, K, and Na content of these samples from the two varieties. However, there was a slight, nonsignificant variation in the iron and zinc contents of the samples across the different treatments and maturity stages.

**TABLE 11:** Micronutrient composition of selected samples of the two *S. aethiopicum* varieties.

Samples	Parameters (mg/100 g)						
	P	Ca	Mg	Fe	Zn	K	Na
PVV1	ND	ND	ND	ND	ND	ND	ND
REV1	356.52 ± 0.04^g^	647 ± 1.41^g^	17.98 ± 0.03^d^	2.38 ± 0.01^g^	9.91 ± 0.01^c^	1248.90 ± 0.20^c^	71.24 ± 0.34^c^
REBV1	697.39 ± 0.38^a^	1372.5 ± 4.95^b^	13.06 ± 0.09^f^	9.08 ± 0.14^a^	21.97 ± 0.32^b^	389.27 ± 0.59^h^	34.05 ± 0.08^g^
VEBV1	68.26 ± 0.08^i^	1150.5 ± 2.12^c^	12.63 ± 0.01^g^	2.27 ± 0.01^g^	2.17 ± 0.01^f^	494.09 ± 0.09^g^	40.51 ± 0.01^f^
REVV1	492.94 ± 0.08^e^	733 ± 4.24^f^	22.84 ± 0.03^b^	7.37 ± 0.16^b^	4.21 ± 0.02^e^	1248.34 ± 0.35^d^	80.27 ± 0.40^b^
RSPBV1	77.07 ± 0.01^h^	1070.5 ± 2.12^d^	16.51 ± 0.02^e^	5.83 ± 0.01^d^	6.47 ± 0.01^d^	642.78 ± 0.01^f^	47.45 ± 0.01^e^
VSPVV1	521.67 ± 0.10^c^	486.5 ± 2.12^i^	13.06 ± 0.09^f^	4.64 ± 0.01^e^	9.92 ± 0.01^c^	494.19 ± 0.78^g^	40.51 ± 0.01^f^
PVV2	ND	ND	ND	ND	ND	ND	ND
VEVV2	376.36 ± 0.40^f^	511 ± 1.41^h^	19.22 ± 0.32^c^	3.51 ± 0.01^f^	27.07 ± 0.10^a^	1078.59 ± 0.01^e^	62.93 ± 0.01^d^
VSPBV2	561.10 ± 0.16^b^	922.5 ± 3.54^e^	22.82 ± 0.03^b^	7.48 ± 0.03^b^	1.02 ± 0.01^g^	1527.07 ± 0.07^b^	90.076 ± 0.11^a^
VSPVV2	426.13 ± 0.78^f^	326.3 ± 3.6^e^	23.34 ± 0.04^b^	3.12 ± 0.01^g^	6.34 ± 0.01^d^	345.76 ± 0.86^h^	34.56 ± 0.01^f^

*Note:* Values with different letters in the same column are significantly different (*p* < 0.05). PVV1 = cortex of green fruit boiled and steamed Variety 1, REBV1 = skin‐on red boiled Fruit Variety 1, VEBV1 = skin‐on green boiled Fruit Variety 1, VEVV1 = skin‐on green steamed Fruit Variety 1, RSPBV1 = pulp of red boiled Fruit Variety 1, VSPBV1 = pulp of green boiled Fruit Variety 1, VSPVV1 = pulp of green steamed Fruit Variety 1, PVV2 = cortex of green fruit boiled and steamed Variety 2, VEVV2 = skin‐on green steamed Fruit Variety 2, VSPBV2 = pulp of boiled green Fruit Variety V2, VSPVV2 = pulp of steamed green Fruit Variety V2, P = phosphorus, Ca = calcium, Mg = magnesium, Fe = iron, Zn = zinc, K = potassium, and Na = sodium.

Abbreviation: ND = not determined.

#### 3.1.8. In Vitro Evaluation of the Alpha‐Amylase Inhibition Capacity of Selected Samples

**FIGURE 7:**
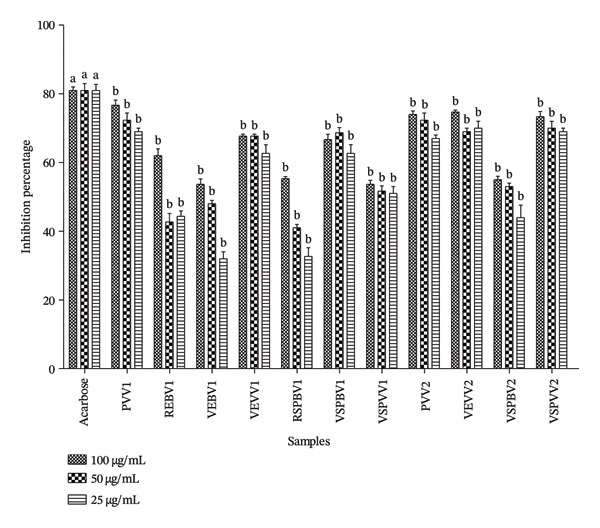
Alpha‐amylase inhibition capacity of the different *Solanum aethiopicum* samples. PVV1 = cortex of green fruit boiled and steamed Variety 1, REBV1 = skin‐on red boiled Fruit Variety 1, VEBV1 = skin‐on green boiled Fruit Variety 1, VEVV1 = skin‐on green steamed Fruit Variety 1, RSPBV1 = pulp of red boiled Fruit Variety 1, VSPBV1 = pulp of green boiled Fruit Variety 1, VSPVV1 = pulp of green steamed Fruit Variety 1, PVV2 = cortex of green fruit boiled and steamed Variety 2, VEVV2 = skin‐on green steamed Fruit Variety 2, VSPBV2 = pulp of boiled green Fruit Variety V2, and VSPVV2 = pulp of steamed green Fruit Variety V2.

The results of α‐amylase inhibition of the hydroethanol extracts of *S. aethiopicum* are presented in Figure 7. It can be seen that the lowest percentage of inhibition was observed in the PVV1 sample, remaining below 50% at all concentrations. The figure also shows that the samples VEVV1, VSPBV1, PVV2, VEVV2, and VSPVV2 had higher inhibition percentages, exceeding 50%.

#### 3.1.9. In Vivo Evaluation of the Hypoglycemic Activity of *S. aethiopicum* Extracts Using the OGTT

Figure 8 shows the results of the OGTT of *S. aethiopicum* extracts. In male rats, after administration of the extracts, there was no increase in peak levels with the RSPBV1 sample, indicating spontaneous regulation. A similar result was obtained with the PVV1 and RSPBV1 samples in female rats. In general, blood glucose peaks were lower in female rats than in male rats. For example, at 30 min, blood glucose peaks in male test rats ranged from 84 mg/dL (PSPBV2) to 147 mg/dL (PSPVV1), while in female rats, the range was from 77 mg/dL (PVV1) to 142 mg/dL (VEVV1). The figure also shows that after administration of the treatments and the D‐glucose solution, all groups experienced an increase in peak blood glucose levels within 30 min. Regulation of blood glucose levels began 30 min after.

FIGURE 8Hypoglycemic capacities of the hydroethanol extracts of the different samples of *Solanum aethiopicum* in male (a) and female (b) rats. PVV1 = cortex of green fruit boiled and steamed Variety 1, REBV1 = skin‐on red boiled Fruit Variety 1, VEBV1 = skin‐on green boiled Fruit Variety 1, VEVV1 = skin‐on green steamed Fruit Variety 1, RSPBV1 = pulp of red boiled Fruit Variety 1, VSPBV1 = pulp of green boiled Fruit Variety 1, VSPVV1 = pulp of green steamed Fruit Variety 1, PVV2 = cortex of green fruit boiled and steamed Variety 2, VEVV2 = skin‐on green steamed Fruit Variety 2, VSPBV2 = pulp of boiled green Fruit Variety V2, VSPVV2 = pulp of steamed green Fruit Variety V2, TN = negative control, TNe = neutral control, and TTM = metformin‐treated control.

**Figure:**
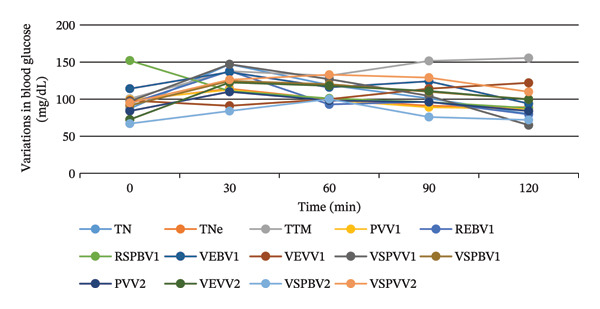
(a)

**Figure:**
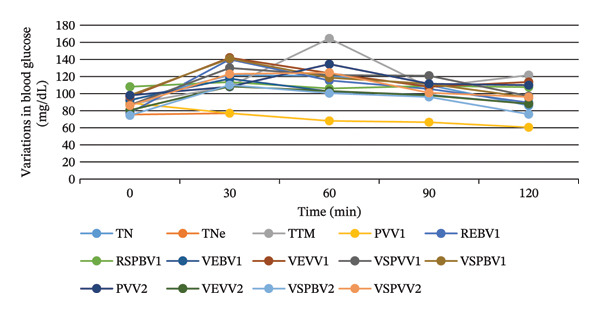
(b)

### 3.2. Discussion

The effect of the pigmentation of the different varieties on the content of some antinutritional compounds showed in general that the phytate, oxalate, and tannin contents fell after the treatments for all varieties. Some traditional methods and technological processing, such as roasting, cooking, soaking, milling, debranning, germination, and fermentation, have been identified as means to reduce antinutrients in foods. During heat treatments, there is rupture of the cell wall of plants, thus leaching out the soluble antinutrients [33]. From a nutritional point of view, this drop in antinutritional compounds is a good sign, as it has been established that antinutritional compounds have the capacity to reduce the bioavailability of nutrients [34]. These results were similar to those of Fitriani et al. [35] who found that steaming, boiling, and frying reduce the antinutrient content, such as phytic acid, tannins, and trypsin inhibitor in Kabau (*Archidendron bubalinum*).

Proximate and mineral composition revealed different values in protein, lipid, and fiber depending on the variety, ripeness stage, and culinary treatments. For example, the lowest fiber content was 14.29%DM, corresponding to whole red fruit of variety 1 (REV1), while the highest value was 43.34%DM, corresponding to green fruit pulp of variety 2 (PVV2). It is evident from previous studies that the maturity stage significantly influences the nutritional and phytochemical composition of fruits, and the content may increase or decrease at different stages of maturity [36]. The results of this study are in line with those of Srivastava et al. [37], who found that protein, fiber, and ash content of wood apple (*Feronia limonia* (L.) Swingle) declined by 44.7%, 47.3%, and 18.16%, respectively, as full ripening progressed. However, Diba et al. [38] found in their work with fig fruits that the late maturity stage resulted in the highest crude fiber content. Authors have reported three health benefits of fiber: lowering cholesterol levels, improving glycemic control, and normalizing stool consistency. These effects are due to the viscosity of soluble fibers. It has been demonstrated that nonviscous soluble fibers and insoluble fibers do not provide these viscosity‐dependent health benefits, whereas high‐viscosity fibers exhibit significant effects on cholesterol lowering and improved glycemic control [39]. The high fiber content in the varieties of *S. aethiopicum* used in this study indicates that consumption of these fruits may be beneficial for blood sugar regulation.

The micronutrient results showed a significant difference (*p* < 0.05) between the different samples with the treatments applied. The iron, zinc, magnesium, potassium, and sodium contents ranged, respectively, between 2.27 ± 0.01 and 7.48 ± 0.03 mg/100 g; 9.91 ± 0.01 and 27.07 ± 0.10 mg/100 g; 12.63 ± 0.01 and 23.34 ± 0.04 mg/100 g; 345.76 ± 0.86 and 1527.07 ± 0.07 mg/100 g; and 34.05 ± 0.08 and 90.076 ± 0.11 mg/100 g. The optimal functioning of the immune system depends on the presence of minerals in food intake. A deficiency in certain minerals, such as magnesium, zinc, copper, iron, and selenium, could temporarily, in the long term, reduce immune competence or even disrupt systemic inflammation regulation [40].

The results of the total phenol and flavonoid contents of the aqueous extracts of powders of the two varieties of *S. aethiopicum* associated with the different treatments and the state of maturity of the fruits show that the total phenol content was high in the untreated red samples (REV1 and REV2) in the two varieties (101.07 and 147.97 mg GAE/g, respectively). This could lead to the conclusion that the effect of pigmentation has a significant influence on polyphenol levels in *S. aethiopicum* fruits. This is in line with the results of Staveckiene and collaborators [41] who demonstrate that the stage of ripening and the species of Solanum fruits influence the accumulation of polyphenols and antioxidant activity. The greatest antioxidant activity was found at ripening stage I, while the highest content of total phenolic acid was found at ripening stage III. This is due to the fact that factors, such as analytical techniques, cultivar differences, and growing conditions that influence the accumulation of phenolic acids in fruits, are influenced by the ripening stage [42–44]. The results also indicate that both cooking methods have a negative impact on the total phenol content of *S. aethiopicum* fruit. This decrease could be explained by the great ease with which polyphenols are extracted from cooked samples, following a strong embrittlement of the cell walls of plant tissues by heat, which can lead to the leaching of these compounds and result in their loss [45].

The polar coordinates (*L* ^∗^ *C* ^∗^ *h* ^∗^) of the basic color parameters were presented to establish a link between the pigmentation of the various samples selected and the physiology of diabetes in general, including type 2 diabetes in particular. According to the literature, the constant parameter *L* ^∗^ circumscribes a luminosity value ranging from black (0) to white (100), indicating the concentration of dark pigments, such as anthocyanins. That said, the samples of the green variety that showed a luminosity value above 50 for all the cooking treatments (i.e., VSPBV2, VEBV1, VSPBV1, and PVV1) would indicate that they were richer in these bioactive elements, which are responsible for the brilliance of the sample and probably for its acceptability to consumers. As for *C* ^∗^ representing color intensity, a higher value generally indicates a more saturated or more intense color for the sample of interest, in this case, a higher content of the main designated bioactive compounds [46]. In general terms, this means that of the 11 samples recommended for better management of patients with type II diabetes, VSPBV2 and RSPBV1 have the highest anthocyanin content. The value of *h* ^∗^, which is representative of hue, i.e., the shade of color expressed in degrees on a chromatic circle ranging from red (0°) to yellow (90°), green (180°), and blue (270°), can reveal the types of pigments present, such as carotenoids (yellow–orange) and/or anthocyanins (red–violet). The PVV2, VEVV1, and VSPBV2 samples, which showed values tending toward 90°, would probably indicate the same levels of carotenoid pigments. Thus, due to the higher luminosity of the green eggplant, we would recommend variety 1, keeping the eggplant pulps and boiling. As for chromaticity, boiling is still the preferred method, but variety 2 (red), preferably without the pulps, is much more popular. As for the intensity of the various pigment contents, the results tend to support steaming to increase the carotenoid content, which was found to be higher in the green samples. The hue was more likely to be closer to 180° for the green eggplant and closer to 0° for the red eggplant, revealing different pigment types as mentioned. These differences in pigment composition are thought to be linearly related to the antioxidant and anti‐inflammatory properties of eggplants, which are beneficial in preventing type II diabetes. Thus, the analysis of *L* ^∗^ *C* ^∗^ *h* ^∗^ color parameters can provide valuable information on the bioactive pigment content of eggplant fruits, helping to better understand their beneficial properties for health and the prevention of type II diabetes [47, 48].

The presence of 29 bioactive compounds, mainly represented in variety V1, and 24 compounds in variety V2 of *S. aethiopicum* was revealed by the GC–MS method. These results were in line with those of Varsha et al. [49] where the study revealed the presence of 17 and 14 bioactive compounds from methanol extracts of *Garcinia cambogia* and *Garcinia indica*, respectively. The difference in the bioactive compound numbers found in different varieties of plant depends on factors, such as climate, soil, water core, genotypes, season, harvest date, geographic region, storage, bitter pit, and irradiation, as well as other conditions [50]. However, genetic variations were recognized as key factors that affect the contents of active constituents [51]. Among these compounds, certain ones can possess potential effects on blood sugar regulation and antioxidant properties, such as stigmasterol, gamma.‐sitosterol, catechol, 6‐methyl 4H‐pyran‐4‐one; 2,3‐dihydro‐3,5‐dihydroxy [52–55].

The above information allows us to draw conclusions about the antioxidant and antidiabetic properties of *S. aethiopicum*. One of the most important mechanisms of action of antioxidants is the scavenging of reactive oxygen species and free radicals. DPPH forms a stable molecule by accepting an electron or proton and is therefore used to determine the radical scavenging effect of natural products. Also, Sridhar and Charles [56] showed that the DPPH radical has a dark purple hue in solution, but when reduced into DPPH‐H, it turns colorless or light yellow. The IC_50_ DPPH results for the aqueous extracts of the different powders in the samples show that the REV1 and REV2 samples have a high antioxidant activity (IC_50_ DPPH = 17.58 and 12.79 μg/mL) compared with all other samples which have a moderate antioxidant capacity. This could be due to the nontreatment applied to the REV1 and REV2 samples. The classification of Souri et al. [57] indicates that when the IC_50_ value is < 20 μg/mL, antioxidant activity is high; when it is between 20 μg/mL < IC_50_ < 75 μg/mL, antioxidant activity is moderate; and when the IC_50_ > 75 μg/mL, antioxidant activity is low. Based on this classification, it is clear that *S. aethiopicum* samples have both moderate and high antioxidant activity depending on culinary treatment, variety, and ripeness. The reducing power of *S. aethiopicum* extracts was evaluated in the FRAP test, which represents the concentration of a reducing agent required in 1 g of extract to halve the complex from Fe^3+^ to Fe^2+^. This measurement is important for assessing an antioxidant’s ability to neutralize free radicals, providing an indication of its antioxidant potential. The variation in reducing power recorded in *S. aethiopicum* L. extracts is thought to be due to the variable composition of total phenols and flavonoids in the extracts. There is a correlation between the phenol and flavonoid content and the antioxidant activity of plants [58].

The hypoglycemic capacity of the hydroethanol extracts of the samples from the PCA was evaluated by alpha‐amylase inhibition tests in vitro. This enzyme hydrolyzes α‐1.4 glycosidic bonds in polysaccharides to form low‐molecular‐weight molecules, such as disaccharides and trisaccharides [59, 60]. The percentages of inhibition obtained for all samples were above 50%, except for sample PVV1, which showed inhibition below 50% at all concentrations. These results are consistent with the work of Ponticelli et al. [9], who reported that *S. aethiopicum* has a strong capacity to inhibit α‐amylase activity, thereby reducing intestinal absorption of postprandial glucose. The hypoglycemic effect was also observed in the OGTT. It should be noted that these extracts regulated blood glucose levels within 30 min after the postprandial rise in all samples, compared with the control groups, which showed a delayed regulation. This demonstrates the ability of these extracts to regulate postprandial glycemia rapidly. Blood glucose peaks were lower in female rats than in male rats, and this could be due to hormonal factors. Kang et al. [61] reported that xenoestrogens can reverse the reduction in insulin and the mRNA expression of insulin transcriptional regulators of pancreatic islet beta cells induced by streptozotocin, and this effect is related to the activation of NF‐kB, which produces anti‐apoptotic effects. This shows the protective role of estrogens against the action of streptozotocin, supporting the idea that female rats are less sensitive to the action of streptozotocin than males.

In view of the nutritional composition and biological properties revealed by these two *S. aethiopicum* varieties, they could be considered functional foods or used in the formulation of foods for therapeutic purposes. These properties would be linked to a number of factors, such as phenolic compound content, dietary fiber content, and chemical compounds detected by GC–MS. However, it would be interesting in future studies to determine the portion of soluble and insoluble fibers in the samples, to really study the mechanisms of action of chemical compounds on the biological properties observed, and to formulate a therapeutic food based on *S. aethiopicum* fruits. Also, although cortex samples are known for their fibrous nature, they have not been analyzed separately for each cooking method to have information in their specific nutritional and chemical composition. As GC–MS analyses were only performed on raw samples at the green maturity stage of each variety, it would be interesting to know the bioactive compounds present in eggplant based on all maturity stages and culinary treatments.

## 4. Conclusion

The aim of this study was to characterize two varieties of *S. aethiopicum* in terms of their ripeness and culinary treatments to propose the most suitable one for controlling hyperglycemia. Antinutrient levels have decreased significantly, especially in red fruits. Regardless of variety and ripeness, total phenolic and dietary fiber content decreased with culinary processing, and the effects were more pronounced with the modern variety. The antioxidant activities of the samples were found to be very high in the untreated samples and moderate in the treated samples. Consequently, the inhibition percentage of alpha‐amylase in all extracts was above 50%, proving that Solanum fruits consumed raw or cooked have the ability to regulate blood sugar levels. The GC–MS revealed the presence of 29 phytochemical compounds, mainly represented in variety V1, and 24 in variety V2. In conclusion, the whole red steamed fruits of the local variety of *S. aethiopicum* exhibited the best nutritional, antioxidant, and hypoglycemic properties.

## Author Contributions

Ghislain Maffo Tazoho: conceptualization, data curation, formal analysis, investigation, methodology, resources, software, supervision, validation, visualization, and writing–original draft. Josephat Gabriel Zazi Weike: data curation, formal analysis, investigation, methodology, resources, software, validation, and writing–original draft. Justine Odelonne Kenfack: investigation, methodology, resources, validation, and writing–review and editing. Vanis Slauvers Akago: investigation, methodology, resources, validation, and writing–review and editing. Donald Sévérin Dangang Bossi: methodology, resources, software, supervision, validation, visualization, and writing–review and editing. Hermine Doungue Tsafack: methodology, resources, software, supervision, validation, visualization, and writing–review and editing. Hilaire Macaire Womeni: methodology, project administration, resources, validation, and writing–review and editing. Inocent Gouado: methodology, project administration, resources, validation, and writing–review and editing.

## Funding

No funding was received.

## Conflicts of Interest

The authors declare no conflicts of interest.

## Data Availability

The data that support the findings of this study are available from the corresponding author upon reasonable request.
